# Functional work disability from the perspectives of persons with systemic lupus erythematosus: a qualitative thematic analysis

**DOI:** 10.1186/s13075-025-03572-1

**Published:** 2025-05-26

**Authors:** Behdin Nowrouzi-Kia, Aaron S. Howe, Anson Li, Jeremy Tan, Natalia Saade-Cleves, Kevon Jules, Malak Sadek, Ali Bani-Fatemi, Antonio Avina-Zubieta, Mary T. Fox, William Shaw, Derek Haaland, Janet Pope, Paul R. Fortin, Kathleen S. Bingham, Christine Peschken, Jennifer Reynolds, Catherine Ivory, Dafna D. Gladman, Murray B. Urowitz, Jorge Sanchez-Guerrero, Lily S. H. Lim, Stephanie Keeling, Patti Katz, Mahta Kavkan, Dennisse Bonilla, Wils Nielsen, Zahi Touma

**Affiliations:** 1 ReSTORE Lab, Department of Occupational Science and Occupational Therapy, Temerty Faculty of Medicine, University of Toronto, 500 University Avenue, Toronto, ON M5G 1V7 Canada; 2 Krembil Research Institute-University Health Network, 60 Leonard Avenue, Toronto, ON M5T 0S8 Canada; 3 Centre for Research in Occupational Safety & Health, Laurentian University, 935 Ramsey Lake Rd, Sudbury, ON P3E 2C6 Canada; 4 University of Toronto, Toronto, ON Canada; 5 University of Western Ontario, London, ON Canada; 6 University of British Columbia / Arthritis Research Canada, British Colombia, Canada; 7 School of Nursing, York University, Toronto, ON Canada; 8 School of Medicine, University of Connecticut, Farmington, CT USA; 9 The Waterside Clinic, Barrie, ON Canada; 10 St. Joseph’s Health Care, London, ON Canada; 11 Centre de recherche du CHU Québec, Université Laval (CHUL), Québec, Québec Canada; 12 Centre for Mental Health, University Health Network, Toronto, ON Canada; 13 Department of Psychiatry, University of Toronto, Toronto, Canada; 14 University of Manitoba, Winnipeg, MB Canada; 15 Mary Pack Arthritis Program, Vancouver Arthritis Centre, Vancouver, BC Canada; 16 The Ottawa Hospital Arthritis Centre, Division of Rheumatology, Ottawa, ON Canada; 17 Schroeder Arthritis Institute, Krembil Research Institute, University Health Network, Toronto, Canada; 18 Centre for Prognosis Studies in Rheumatic Diseases, University of Toronto Lupus Clinic, Toronto Western Hospital, Toronto, Canada; 19 Division of Rheumatology, University of Alberta, Edmonton, AB Canada; 20 University of California, San Francisco, San Francisco, USA

**Keywords:** Disease-related outcomes, Employment history, Health-related quality of life, Lived experiences, Mental well-being, Multidisciplinary interventions, Systemic lupus erythematosus, Work disability

## Abstract

**Background:**

Systemic lupus erythematosus (SLE) disease symptoms that can significantly restrict work ability and work participation resulting in reduced mental well-being. This study investigates the significant impact of work participation and disability on the mental wellbeing, health-related quality of life, and disease-related outcomes in individuals with SLE.

**Methods:**

With the objective of creating an SLE-related functional profile rooted in work disability (WD) prevention, 46 SLE patients were purposively recruited from Canadian medical centres. Through semi-structured interviews guided by a WD prevention framework, factors associated with WD and lived experiences of SLE-related WD were qualitatively explored. Braun and Clarke’s six-stage inductive thematic analysis was used to organize the data.

**Results:**

Most participants experienced some form of work disability across their employment history related to their clinical manifestations of SLE, including hospitalizations, physical limitations, fatigue, and neurocognitive symptoms (e.g. brain fog). Thematic analysis revealed three key themes: (a) the influence of illness experience on work, (b) the stigmatization of illness disclosure, and (c) the availability of workplace resources/accommodations. Participants emphasized the desirability of work with reduced physical and mental demands, increased personal control, and workplace flexibility to prevent WD.

**Conclusion:**

The study underscores the need for a collaborative, multi-component, and multidisciplinary intervention targeting psychosocial and workplace factors to establish a goal-oriented preventative framework, potentially improving WD outcomes in SLE individuals.

**Supplementary Information:**

The online version contains supplementary material available at 10.1186/s13075-025-03572-1.

## Background

Systemic lupus erythematosus (SLE) is a chronic and multisystemic autoimmune disease primarily driven by the production of a variety of autoantibodies [1]. It is characterized by its heterogeneous clinical presentation including constitutional symptoms such as fatigue, weight loss, myalgias and fever, as well as skin rashes and arthralgias, cardiac and vascular diseases, pulmonary, central nervous system, and renal involvement [2]. In Canada, the prevalence is 1:1000 with females being 9 times more likely to be affected by SLE than males [3, 4]. Studies describe poorer clinical outcomes in males and higher prevalence rates among Asian, African American, and Hispanic ethnicities [5, 6]. Moreover, people with SLE can present with neuropsychiatric symptoms such as anxiety disorders, cognitive dysfunction, mood disorders, and psychosis that may impact quality of life [7–9]. 

SLE can cause mental and physical functional impairments that affect productivity at work [4]. As such, SLE is considered an important medical cause of work disability (WD) and limitations in functioning [2, 10–12]. WD arises when an individual is incapable to fulfill their job duties, ultimately leading to inability to work, early retirement, sick leave, a change in work hours or responsibilities, and/or a need for work accommodations [13–15]. Limitations of physical and mental functions by SLE may lead to loss of work, which may in turn influence mental well-being and lead to psychological stress, reduced social role participation, anxiety, and depression [16, 17]. Early studies of employment in SLE demonstrate how the disease affects work life, including decreased productivity, reduced working hours, and loss of employment [18]. Results from a systematic review including 9886 individuals found that 32.5% of individuals with SLE experience some form of WD [6]. Prevalence of work loss within 5 years of SLE diagnosis ranges from 15 to 40% and increases to 36% and 51% after 10 and 15 years of the illness, respectively.[4.

]SLE may have profound implications on the ability to engage in productive employment. Many studies have reported the ‘universal’ negative effects of disease activity on employment [20]. Disease symptoms that can significantly restrict work ability include severe pain and swelling, fatigue, reduced mental well-being, and neurocognitive symptoms [19–22]. Studies examining the complications of the physical symptoms in both employed and formerly employed individuals with SLE found that they reported great difficulties in carrying or moving heavy objects, standing or sitting for long periods of time, and working in awkward positions that require crouching, bending, or kneeling [22, 23]. Specifically, Stevens et al. [23] found that 29% of work non-participation was due to lower limb or foot-related symptoms such as episodes of foot swelling, ulcerations, and poor mobility. Although WD was observed across all categories of work, the physical challenges imposed by SLE symptoms had a particularly noticeable impact on the career choices of individuals with SLE who remained in the workforce. Increased prevalence of WD was reported in physically demanding occupations (e.g., operator, fabricator, and labour roles) compared with less physically demanding jobs (e.g. sedentary managerial and professional positions) making accommodations essential for individuals with SLE to remain working in physically challenging jobs.

Based on our review of the literature, it is evident that SLE affects multiple domains of health, work life, and social role participation. Yet, few studies have examined WD from a multidimensional perspective utilizing the patient’s illness experiences. To address this gap, our thematic analysis of interviews builds upon existing literature on WD prevention frameworks, demographic variables, and symptomatic manifestations among SLE patients. This study explores the roles and interactions between an SLE patient’s workplace, healthcare, personal, and compensation systems, in contributing to and mitigating WD.

## Methods

### Sample recruitment

The current study was part of a larger study utilising a mixed-methods sequential explanatory approach, including a cross-sectional survey. Data collection was done at 12 sites across Canada where 50 participants were recruited. All participants were seen during regular visits to the 12 centres (11 teaching hospitals and one community centre) and were approached to take part in a semi-structured research interview. Eligible participants met the ACR classification revised criteria [24] or the EULAR/ACR classification criteria SLE [25], were between the ages of 18–65, and proficient in English. The survey included socio-demographic questions related to work functioning, including the number of years of work experience, whether they do shift work, typical hours worked, and working arrangement. Several clinical measures, including the SLEDAI-2 K, SLICC/ACR Damage Index (SDI), EULAR/ACR 2019 classification criteria form and current medication (e.g., glucocorticoids, anti-malarial, and immunosuppressants) were collected. The University Health Network Research Ethics Board and the University of Toronto Research Ethics Board reviewed and approved this study. Participants provided written informed consent and research activities were conducted in accordance with the Declaration of Helsinki.

### Study framework

Examining WD and function in SLE means recognising the complex, multidimensional, and temporal dimensions of SLE and its relationship to work, warranting a multidisciplinary perspective and approach to WD management. This study employs the WD prevention framework that describes disability in the workplace as a result not only of the workers’ characteristics, but also environmental factors [13, 26]. The framework also indicates personal, workplace, healthcare, and compensation systems are influential to a worker’s health and well-being. Personal systems are comprised of personal experiences of physical, cognitive, and psychosocial elements, while workplace systems include work environment, organisation, department, and job factors and elements. These systems interact with healthcare (inter-/intradisciplinary and organisational teams, physicians, and other healthcare professionals) and compensation (legal and financial) systems to influence the experience of illness, injury, and disability at the individual level. Aligned with the goals of this study, the WD prevention model acknowledges that such factors must be recognised and managed if successful disability prevention is to be achieved. This framework was used to inform development of the interview questions and subsequent probing questions during the interview process to stimulate a rich understanding of WD in SLE.

### Interview procedures

Semi-structured interviews were conducted to qualitatively identify factors associated with WD and explore lived experiences of SLE-related WD across participants’ employment history. Interview content was developed based on a WD prevention framework. Questions posed were constructed to capture the experience of working with SLE, challenges perceived, coping mechanisms, assistance from supervisors, and impact in life outside of work (refer to Appendix A). The interviews were conducted throughout July 2021 to July 2023 by trained MA and PhD interviewers (WN, ASH, BN-K). The interviews focused on current or past employment, year of diagnosis, difficulties on work due to SLE, coping mechanisms, mental and physical symptoms experienced, support from managers, among others. Rapport was established with each participant prior to discussing the interview questions. The session began by making the participant comfortable through informal conversation, including confirmation of their name and brief work history. The interviews, which were held via phone, were audio recorded and lasted 30–40 min in duration. Repeat interviews were not conducted. Interview sample size was determined through exploratory sampling of individuals with SLE nationally and thematic data saturation (e.g. emergent new themes and participant response rate) [27, 28]. 

### Qualitative analysis

Thematic analysis was performed using the guiding principles outlined by Braun and Clarke six-stage inductive thematic analysis [29]. Qualitative thematic analysis was performed using NVivo 12.7. The multidisciplinary research team was comprised of undergraduate trainees, PhD students, and post-graduate research analysts and the primary author (ASH, AL, JT, NS-C, KJ, MS, AB-F, BN-K) each of which individually reviewed all transcripts and were involved in the data coding process. *Stage 1*: Each research team member familiarized themselves with the transcripts and subsequently performed independent open coding of key themes and concepts identified to discuss with the research team during weekly research meetings. Each team member kept their own reflexive notes regarding the interpretations of the data. *Stage 3*: A reflexive approach was employed to review open coding findings within the research team to identify different perspectives and qualitative organisation of the data. After open coding 25% of the transcripts, research meetings were held to develop a codebook that would guide the analysis. Debriefing of the preliminary themes and established codes were performed bi-weekly to discuss data saturation and the need for further data collection. *Stage 4 and 5*: Coding and re-examination of preliminary themes were completed until data saturation was achieved. Final review and consultation of the coding and thematic development was evaluated by two authors with clinical experience in rheumatology and rehabilitation settings (BN-K, ZT). *Stage 6*: Examination of the existing SLE and WD literature was performed to structure the findings.

### Methodological trustworthiness and rigor

Qualitative analysis included multiple methods of methodological rigour including triangulation of coding, direct quotes to describe results, and verification of accurate transcription of the audio recorded interview. Additional research triangulation was performed when consulting co-authors familiar with the WD prevention framework and quality of life management in occupational and rheumatology settings in thematic refinement and write-up. Reflections regarding the interviewers’ insights and impressions of the data were gathered and detailed in interview notes. Communication among the multi-disciplinary team was documented during meetings and email exchanges to ensure an audit trail and established historical record of agreement between all members of the research team. Independent reflexive journaling during transcription verification, open coding, and thematic development was maintained by each author involved in the qualitative analysis. The study followed the reporting structure of the COnsolidated criteria for REporting Qualitative research (COREQ) Checklist.

## Results

**Fig. 1:**
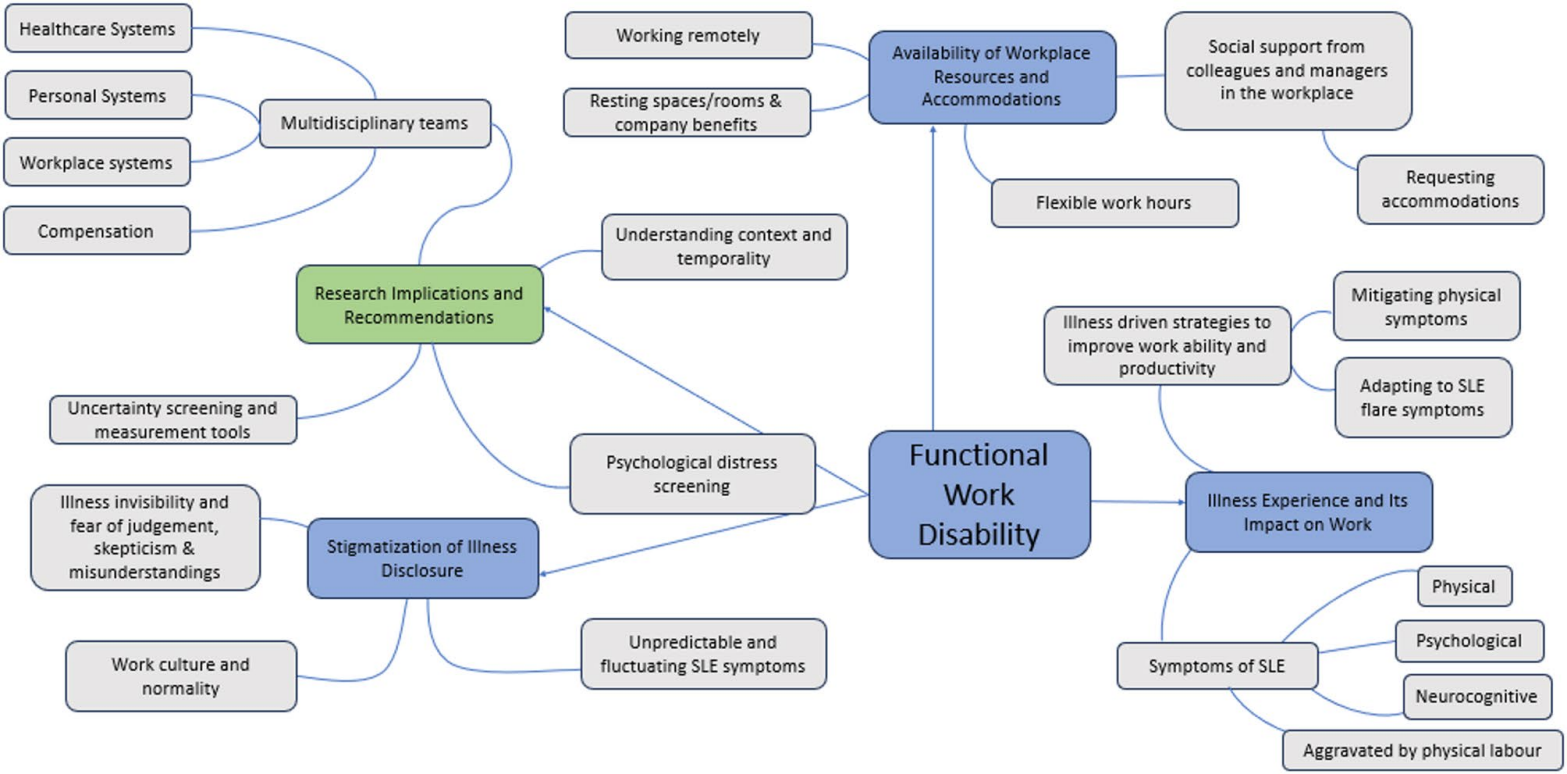
Concept map detailing themes and their interconnections

The final sample included 46 individuals previously or currently employed and living with SLE. Four participants did not respond to the invitation to participate in the semi-structured interview. Participant characteristics including demographic variables, clinical variables, employment activities, and workplace accommodations (Table 1). The majority of participants were female (91.3%) with a mean age of 45.3 ± 12.2 years. Some participants reported working part-time, casual or performed non-traditional work arrangements (e.g. seasonal, temporary). Most participants experienced some form of WD across their employment history related to their clinical manifestations of SLE, including hospitalizations, physical limitations, fatigue, and neurocognitive symptoms (e.g. brain fog). Qualitative data was organized into three themes: (1) illness experience and its impact on work, (2) stigma of disclosure, and (3) available resources and accommodations. These themes were conceptualized within the WD prevention framework as shown in Fig. 1.

**Table 1:** Participant characteristics

	Participants (*n* = 46)
Age, years (SD)	45.3 (12.2)
Mean age of SLE diagnosis (SD), years (SD)	27.6 (12.3)
Sex, N (%)	Female: 42 (91.3%) Male: 4 (8.7%)
Race, N (%)	Caucasian: 30 (65.2%) Black: 5 (10.9%) Filipino: 4 (8.7%) Mixed Race: 3 (6.5%) Chinese: 2 (4.3%) Other: 2 (4.3%)
Education, N (%)	University: 21 (45.7%) College: 13 (28.3%) High School: 10 (21.7%) Not answered: 2 (4.3%)
Marital Status, N (%)	Single: 22 (47.8%) Married: 15 (32.6%) Common Law: 6 (13.0%) Separated: 2 (4.3%) Not answered: 1 (2.2%)
Employment status N (%)	Employed: 29 (63.0%) Self-employed: 6 (13.0%) Retired: 5 (10.9%) Unemployed: 2 (4.3%) Sick leave: 1 (2.2%) Long term disability: 1 (2.2%) Student: 1 (2.2%) Not answered: 1 (2.2%)
Employment activities, N (%)	Sedentary: 30 (56.6%) Mixed: 5 (9.4%) Manual: 18 (34.0%)
Workplace accommodations, N (%)	Flexible schedule/breaks: 56 (51.4%) Physical assistive aids: 32 (29.4%) Increased time off: 21 (19.3%)
Corticosteroid use	Yes: 19 (41.3%) No: 27 (58.7%)
Mean prednisone dose	7.0 mg/d (range: 1–20 mg)
Mean SLEDAI (SD)	3.5 (3.7) (range: 0–14)
Mean SDI (SD)	1.1 (1.5) (range: 0–6)

### Theme 1: the illness experience and its impact on work

Participants reported experiencing different physical, psychological, and cognitive symptoms which made their work very challenging and had negative impacts on their health-related quality of life. Most participants reported that fatigue ranked among the most challenging symptoms associated with working with SLE. The most difficult aspect of their day resided in their morning routine and preparing for work, hindered by fatigue that impeded their ability to get out of bed. Additionally, fatigue made commuting to work more difficult especially during the winter months.

Another common symptom was pain experienced in the joints and bone. Joint pain was reported to decrease working time as participants found working for prolonged periods of time without breaks very challenging. For example, participants mentioned that they cannot type on a keyboard in a certain position or sit and stand for a prolonged period. Moreover, most participants reported that due to joint pain they need to be mobile as they cannot tolerate staying in prolonged positions, which limits their ability in working sedentary jobs. Several participants also mentioned that their fingers and toes would swell and turn purple or blue unpredictably resulting in physical limitations (e.g. inability to stand or sit for prolonged periods of time, difficulty performing work activities with their hands, challenges with lifting objects, difficulty feeling sizes, shapes, and textures) during work. Many of the occupations reported required prolonged sitting and the participants reported often struggling with frequent flare ups, such as swelling of limbs or joint pains. Mental and psychological strain was experienced due to these symptom manifestations as many participants reported feelings of worry and judgement if customers, clients, or co-workers observed their hands or feet.

Several participants also reported that SLE had negatively impacted their mental health. Some participants mentioned that they were struggling more in terms of their mental health than physical health. Feelings of depression due to a perceived inability to achieve one’s aspirations and to do things they wanted to do at work or in their personal life were reported. For example, participants often compared themselves to their co-workers who had received better opportunities and promotions and reported feelings of anger and depression viewing their illness as an obstacle. Consequently, some felt unmotivated and lacked confidence. Other participants reported experiencing high levels of stress and anxiety. For example, they reported that the unpredictability of SLE flare ups was a source of compounding mental and psychosocial stress. Another source of stress for some participants was the lack of mental health support. Deciding to leave work or have an early retirement due to feelings of being overwhelmed by flare ups of SLE symptoms exacerbated financial stress and anxiety about their future.

Few participants mentioned that SLE affected their memory and cognitive function. They reported forgetfulness and inability to concentrate requiring use of adaptive coping strategies including pacing, to-do lists, written notes, and phone reminders. Some participants reported brain fog that would manifest in difficulty with word finding, slow processing speed, and sustaining attention. The mental and physical aspects of the illness experience limited participants’ working hours and productivity. For example, physical labour was challenging for many participants. Most participants reported that symptom aggravation from physical labour did not affect their performance, but rather it affected their presenteeism and absenteeism. For example, many participants during a disease flare or symptom aggravation had to take prolonged sick leave, unpaid leaves of absence, and potentially consider resignation from their employment. Some participants were adversely affected by absenteeism when it came to their annual review and evaluation as they got negative reviews about their work performance and commitment. Due to these challenges, many participants reported ambivalence about returning to work after their sick leave. Others experienced fear of using sick leave or disability benefits and continued to work through significant mental and physical limitations to avoid loss of employment and financial insecurity.

#### Subtheme 1.1: illness driven strategies to improve work ability and productivity

The ability to manage the symptoms of their chronic illness was identified as an essential factor in maintaining work ability and a sense of productivity within their occupation. The prevailing viewpoint among the participants regarding the incorporation of coping mechanisms into their work-life centred on mitigating the physical symptoms associated with SLE. During work, participants reported employing a range of assistive aids to optimise their workspace ergonomics to fit their needs. The most common assistive devices included ergonomic chairs, ergonomic computer mice and keyboards, blue light filters on computer monitors, compression socks and gloves, footstools, and extra layers of clothing. These devices collectively contributed to alleviating pain and swelling of participants’ joints and extremities. To help with the fatigue caused by SLE, participants reported taking advantage of break rooms for short breaks or naps during their work. The ability of the participants to implement these coping mechanisms also provided a sense of control over their work schedule, performance, and environment. The most common coping mechanism used outside the work environment was medication to alleviate lupus flare symptoms. While both non-steroidal anti-inflammatory drugs (NSAIDs) and corticosteroids medications were employed, a subset of participants reported experiencing adverse effects when using corticosteroids, leading them to discontinue their usage. Other less-reported methods of coping with SLE symptoms outside of work include taking warm baths and using medical marijuana.

*P1742: “I will do something like I’ll take a coffee break with a friend*,* or by myself for that matter. Or I do things like manage my environment… This is physical because of the particular symptoms I had like the swelling in the legs. I’ll wear what you call compression socks. So I’ll find kind of ways to control my environment in a way that will lessen some of the symptoms if that makes sense.”*

*P1167: “The bank*,* there were a lot of challenges because it was mainly a standing job so I had to come make it clear to them that I would need to be in a sitting position most of the time*,* and that I needed to have some sort of stool or apparatus there to elevate my legs to prevent swelling up.”*

Alongside managing SLE symptoms when they appear, participants also had to learn to adapt to living with SLE. Most participants acknowledged SLE does present many circumstances that are unexpected, debilitating, and challenging. Optimising their situation required a shift in focus toward their abilities rather than viewing their condition as a hindrance to achieving their goals. The most common approach mentioned by the participants was to have a social network of people who are aware and supportive of their illness and subsequent symptoms, but also an understanding of the participant’s own autonomy as a working individual. This network may include family members, friends, colleagues, supervisors, and healthcare providers. Furthermore, some participants implemented healthier lifestyle changes to pre-emptively reduce the severity of their symptoms flare-ups. For example, meditation, spirituality, physical exercise, and dietary changes away from pro-inflammatory foods were all reported to be used to prepare for work either better mentally or to reduce the flare symptoms experienced. Lastly, some participants mentioned the importance of managing their work around their flare-ups. Although SLE flares may be difficult to predict and not every participant had the benefit of a flexible work schedule, those who were afforded this flexibility strategically aimed to maximize their productivity between flare-ups, allowing them to allocate more time to recover when their symptoms do appear.

*P1803: “Well*,* I think working from home has been a really big blessing because I can do my work at times when I feel very energetic*,* and I don’t need a lot of time. If I’m feeling good*,* I just need 2–3 hours and I’ll be able to do most of my work. So that’s usually in the late evening when I’m feeling a little bit of energy*,* or you know. So that kind of stuff where I’m able to manage my time.”*

### Theme 2. Stigma of disclosure

Illness invisibility can create challenges for participants who considered disclosing their condition to their employer as it has previously led to anecdotes of scepticism, judgment, and misunderstanding from those who are unfamiliar with the SLE’s impact. Some participants were hesitant to disclose their experience of SLE and chronic illnesses to their employers and co-workers. The stigma experienced surrounding the disclosure of SLE appears to manifest from a desire to not be defined solely by their illness when interacting with co-workers and family members in their social roles. While some alluded to not being defined by their illness, they also understood that disclosure could affect how others define them, and in not taking this risk, protects them from potential workplace discrimination or a changed perception of capabilities at work. This protective mechanism is also exhibited where the participant has not yet experienced an episode of discrimination within the workplace, reflecting a deeper internalization of negative societal perceptions about illness and capability.

*P1167: “Discrimination is a real concern whether I decide to or decide not to disclose. I’m always thinking*,* ‘what is that person going to think of me?’*,* or potentially because some people may be supportive and others not*,* right?… When it comes to work*,* I’ve kind of adopted the philosophy that less is more – the less that I tell them*,* the better – though I do provide documentation that I have a disability and especially if I’ve taken a certain amount of sick days or if I’ve maxed out my sick days. But I always try to make sure that whoever is providing that letter doesn’t disclose what the condition is*,* so I do try to refrain from disclosing my illness*,* but I do indicate to them that I do have a disability*,* so I think that’s more to protect me from being discriminated against. It’s not 100% foolproof*,* but in my experience*,* like to be honest with you*,* I don’t think I’ve really had an experience where I feel like I’m being discriminated against in the workplace.”*

Another reason for the reluctance to disclose is the fear of being labelled as “abnormal” or deviating from the norm. Participants often faced the misconception that they should “look sick” to be considered truly ill. Since their symptoms are not readily apparent, they may encounter responses such as, “But you don’t look sick?“, which can undermine their credibility and invalidate their experiences. This creates a sense of shame and embarrassment, as they are put in the position of having to justify their condition to others.

*P1911: “It was really hard for them to understand. At first*,* they said*,* ‘Oh well*,* maybe you shouldn’t work unless you’re that sick*,*‘ or*,* ‘You don’t look too sick.’ It was very hard*,* but they understood it. I just told them they can always look on Google and research – and yes*,* they found that helpful.”*

Lack of understanding contributes to stigma of disclosure, where many people are unfamiliar with the disease and its unpredictable nature. This lack of awareness has led to misconceptions and misunderstandings, with others mistakenly believing that the person is exaggerating or faking their symptoms. The fluctuating symptoms of SLE, which can vary in intensity and unpredictability, further complicate the understanding of the disease. Individuals with SLE may feel well on some days but struggle with debilitating symptoms on others. This inconsistency can be difficult for others to comprehend, making it challenging for participants to explain their condition effectively and may direct others to an external source of research for a better learning experience.

*P110001: “One of the difficulties has been alienation from your fellow workers because they can’t really see what the matter with you is because to most people you look mostly fine.”*

#### Subtheme 2.1: work culture and normality

A few participants stated that disclosure of their illness to employers and coworkers would only be possible and carry less risk once they have achieved success within their work or have proved themselves to be competent assets to their employer.

*P1227: “I absolutely could understand why the vast majority of lupus patients would be concerned about [disclosure]. For me personally*,* it’s been nothing but support - I generate good income for the company*,* I’m an asset to them*,* I’ve built long-term relationships – so I feel I’m unique versus working for a large company that has a number on somebody’s desk and you’re just a dot where you’re going to cost the company money*,* you’re going to cost the company time*,* you’re going to add to their insurance bill. So*,* I can absolutely understand why people with diseases*,* lupus in particular*,* would be concerned about going in and saying*,* ‘Hey I got lupus’*,* and then being ostracized and potentially not taken on to the job… But for myself*,* no*,* I have been absolutely open with my boss at the time who was the first to allow me to do whatever had to be done to make sure I was healthy.”*

*P1742: “When I worked for the architecture firm*,* I did hide it at the beginning from the majority of people. I didn’t want them to think that I am incompetent because of an illness*,* and so once I was able to prove and had validation that my work was good*,* then I was more open about it… Well*,* in the eyes of society*,* I have proven that I can function*,* you know. It’s the same as if you’re a visible minority*,* but there’s this feeling like you need to prove yourself*,* but once you’ve proven yourself*,* then it’s okay to be whatever. It’s not right*,* but I think many people feel like that.”*

Individuals with SLE can derive value from their employment if considered as assets. However, the perceived value of employees in the context of chronic illnesses varies across different work cultures. This perspective suggests that within some work cultures, employees who contribute significantly, generate income, and build lasting relationships can receive support and accommodations to better manage their illness. This reflects a potentially positive aspect of workplace culture, where an employee’s worth is assessed based on their contributions rather than their health status. Conversely, this also implies that in other work environments, SLE participants may face stigma and discrimination if viewed as potential liabilities through increased time to complete assigned job tasks and healthcare costs. In such contexts, disclosure may lead to ostracization, and individuals may be hesitant to reveal their health status to their employers. This fear of being perceived as a financial disruption to the work process can create a culture where concealing one’s health issues becomes the norm.

*P1658: “I have the idea that important employers would want someone that’s healthy – that can go to work all the time. The job itself*,* on top of working hours*,* required us to do overtime*,* so I was hesitant to let her know because this will label me as an unreliable employee if that makes sense. And at that time*,* I also wasn’t permanent*,* so I was striving to get a good standing so that they picked to make me full time basically.”*

Such defeatist attitudes even arise in the case of a student with no prior work history, struggling with a negative internalized perception of their work capability through disability, and the additional burden of having to overcompensate to counteract the devaluation. This can be particularly strong for individuals working to establish themselves in their careers or prove their competence, as they may perceive their health condition as a hindrance to securing job stability, security, and advancement.

*P2017: “I think that I just want to be able to prove myself right now with school and getting good grades to say that ‘Hey I’m competent*,* my grades are great*,* I’m a good student – maybe you should give me a chance. You guys should hire me after*,* even if I do have a disability.’*

Work capability, accessibility, and accommodation play a vital role in rebuilding a sense of normality. Participants expressed that work is not just a means to earn a living but viewed as a crucial part of forming identity and purpose. The ability and opportunity to engage in meaningful employment serves as a pathway to regain control and independence, an area often constrained in SLE. The challenge of intertwining physical and emotional well-being with professional fulfilment is often demanding and desires necessary attention – of patients, healthcare providers, and researchers.

*P2126: “I think as you learn more to help people with lupus*,* every person that has lupus is different*,* and their disease is different. It’s all very individualized*,* right? But to help people as they try to regain their strength and their confidence is to build the transition back into work – and not just work*,* but back into a healthy regular life.”*

### Theme 3. Available resources and accommodations

Working remotely or from home was the most common workplace accommodation provided among participant responses. Working remotely allowed participants to avoid commuting while giving flexibility to allocate time for breaks or to use other coping strategies. Physical accommodations were also valued by participants. Physical accommodations included physical instalments such as a stool or apparatus to elevate legs to prevent swelling up, being excused from tasks that were physically taxing, and protection from UV light (e.g., reducing fluorescent lights or relocating them).

*P1087: “And because I’m not commuting*,* it means that I can go for a walk before work. So*,* it’s breaks during the day*,* but even right before work starts*,* it’s just easier because I’m not commuting.”*

*P1167: “I get to determine how many hours I can handle in a day*,* and I get to say when I need to take a break”*.

*P1658: “She was accommodating in a way that because I was sensitive to light. She was able to ask for some of the fluorescent lights to be turned down or it turned off.”*

*P121933: “I have a standing desk*,* it can stand*,* or it can sit so periodically throughout the day when I’m starting to feel tired*,* I will raise my desk up to standing (…) and I can’t be in the sun.*”

Flexible work hours were also reported to be a helpful workplace accommodation. In these cases, participants were able to work around flare ups by taking breaks when necessary and working at the most efficient times for their needs. This also provided time to utilise coping mechanisms such as taking naps or resting mentally. The next most available work accommodation was coworker support. This included support with mental health or work tasks. Coworker support helped to create a more welcoming and comfortable environment for participants as well.

*P1089: “That was in the case of my current manager. She checks in with me at least once a week. Not just about what’s going on at work*,* but just in general [how] I’m doing. And if there’s too much going on for me*,* she will be like what can I take off your plate?”*

The least available accommodations included resting spaces/rooms, and work or company benefits. Though the mentioned accommodations proved to be beneficial, they were not readily available to every participant. Participants who were not provided with these reported more flexibility in working hours to be their most desired accommodation.

#### Subtheme 3.1: social support from colleagues and managers in the workplace

Regarding the accommodations requested by the participants, different scenarios were commonly distinguished regarding getting them or not. The important determinant for accessible accommodations was contingent on the support given by their supervisor. Some participants expressed that denial of accommodations was due to the fact that there are no official work policies for them. This meant that the arrangements for a better workplace were to be negotiated with the supervisor, and if there was a change of supervisor, the accommodations had to be re-discussed. This also resulted in accommodations that did not adjust to what the person needed, suggesting general adjustments would be made that did not ease the barriers experienced by individuals with SLE at work.

*P1742: “In the university*,* I could ask to have a special chair*,* or not have meetings after a certain time*,* or not to attend meetings in person – there’s that aspect. The university of course has an accommodation process because we have students to accommodate. I never saw the formal accommodation in my workplace – there’s no such thing as a formal accommodation.”*

A change in administrative staff would also imply that the accommodations are rearranged by a new person who might not understand the employee’s needs. According to the participants, the managers who were not supportive showed a lack of empathy towards the employee and therefore, would not be open to make illness-specific adjustments that were requested. Some participants stated that this lack of support could be a misunderstanding of SLE, and the adaptations required to manage symptoms.

*P110001: “They would not give me any of the accommodations that my rheumatologist recommended. Like none of them*,* so because they did not want to set a precedent and I couldn’t tell if they thought that I was faking it or I was too sick to work. It was very stressful because I was not well enough to work full time and my flexibility*,* because there was a change of ownership in the paper and a change of management*,* disappeared.”*

*P1911: “And this new one*,* I pointed out my health condition and he just told me to suck it up.”*

*P1980: “There was one [manager] that wasn’t understanding. I needed time off because I was undergoing a lot of stress at home… I was in an unsafe situation physically and emotionally*,* and the lupus clinic said you should take time off right now because you are at risk*,* by being so stressed*,* of having a flare. I had this note from the lupus clinic*,* I spoke with my rheumatologist - but that manager*,* basically after I had been off for maybe a week or a couple of weeks*,* emailed me and said I wasn’t really sick*,* and I needed to come back to work. So*,* I made the case against her.”*

The inability to get accommodations in many cases led to a job change or dropout from work leading to the creation of work disability. The participants spoke about difficulties of maintaining employment or education that came with the physical restrictions due to SLE flare ups, and unsupportive attitude and commentaries from their supervisors. Some participants discussed this also occurring in educational settings.

P1911: “yeah, if one [manager] was more Understanding I would have stayed.”

*P2053: “The teachers were not very accommodating at all. They were actually very rude about it. Every now and then they would just be like*,* ‘Oh. You showed up late because you had to walk here with a cane? Well*,* screw you.’ I wouldn’t be able to do anything in class.”*

*P2053: “No*,* I never worked. Otherwise*,* I was in school*,* and I dropped out of school and then I started freelancing. I never had a job. I didn’t graduate. I dropped out because of the lupus.”*

When asked about changes or accommodations made at their workplace some participants responded that they did not ask for them. Other participants reported that they opted to push through the symptoms, did not know that it was possible for them or did not need them. There was a shared belief that asking for accommodations might put their job at risk, prevent them from being a successful candidate at an interview, or put them at a disadvantage for career growth opportunities compared to their colleagues. There was also a preference by participants to personalize their accommodations by making their own adjustments as they manage the unpredictability of their symptoms.

*P90048: “Well*,* I’ve had to just accommodate accordingly. But if I’m starting to flare*,* I don’t call in sick – I just continue to work and then when I go home that’s when I take that time to rest up so that I can come back to work the next day.”*

*P1004: “No*,* I had pain*,* but it didn’t interfere with anything. I had to keep pushing.”*

Participants did not feel the need to ask for accommodations when they already had a job that gave them flexible hours, were in remission, or their jobs did not have activities that could exacerbate the symptoms. Even though other participants did feel the need for accommodations, they did not request accommodation when they perceived that the request was not possible due to the nature of their job and their fear of retribution.

*P2017: “Working for a law firm*,* I know it could be very demanding*,* and it’s competitive*,* right? So*,* maybe if I can’t do what they want*,* they can obviously try to find someone else that can do the same amount of work.”*

*P1803: “I think the culture of this kind of work is highly competitive and you have to constantly prove yourself. You have to be on top of everything…you have to be very energetic. I see how other people work*,* right? They’ll be more aggressive and taking on tasks*,* but I don’t really do that and make sure that I can handle what I’m taking on. So*,* these kinds of things I feel like they don’t really work in my favour*,* so I don’t want to ask for more accommodations on top of it.”*

Most participants who received the accommodations they had asked for, also reported having a supportive manager. The participants showed great appreciation for their manager’s understanding their need for accommodations. Support from colleagues, who took on some tasks when participants were experiencing lupus symptoms was also reported to be helpful.

*P90029: “Yeah*,* I had a much more understanding supervisor*,* so if I was in pain*,* she was totally understanding of it… You’re not as stressed*,* you’re not as uptight*,* and you let more things roll off. It was rewarding work because I had supervisors that were very appreciative*,* thankful*,* and encouraging*,* and they didn’t put you down and make you feel bad about yourself*,* so it was a totally different atmosphere how a supportive manager can change and shape the experience for a person.”*

These types of support were cited by participants as motivators for remaining in their job.

## Discussion

The ability to engage in meaningful work plays a fundamental role in shaping personal well-being, health, and overall life satisfaction. Addressing gaps in accessibility to diverse and meaningful work opportunities is crucial in a world where opportunities are unevenly distributed. The current study adds to the existing literature about the impact of SLE on WD and explored challenges of seeking equitable work environments as experienced by participants along their SLE journey. The findings show that many participants experienced a variety of symptomatic manifestations of SLE, stigma in and out of the workplace, and different employer responses when seeking accommodation as they were transitioning out of the COVID-19 pandemic.

Previous studies of WD in SLE and other rheumatic diseases have reported similar findings regarding work context and WD [11, 17, 30]. Work context factors such as reduced workplace support, illness disclosure, greater use of accommodations have been associated with WD [31]. In this study, participants reported that availability of workplace accommodations and psychosocial support at work supported their ability to maintain employment. Modified, flexible hours through working remotely and utilization of sick days were common accommodations utilized to reduce WD and have been shown to be helpful in other studies of individuals living with rheumatic diseases [30, 32, 33]. On the other hand, those that do not have access to work accommodations and experienced many work-related barriers related to their illness driven activity limitations may be at risk for WD and poor work outcomes [34]. Research has shown that illness disclosure and the psychosocial support (e.g. colleague support, supervisor empowerment) may play a critical, protective role in request for accommodations and perception of a positive work environment [12, 35]. For example, a study by Ubhi et al. [36] reported that individuals with SLE attending work without adequate support due fears of job security, illness disclosure difficulties, are more likely to voluntarily leave their workplace. This is confirmed in our findings where many participants reported that a supportive workplace empowered them to continue working and promoted adaptation while performing work duties, especially in the context of the COVID-19 pandemic.

Many participants experienced altered perceptions of their work capability and employability following their diagnosis [33, 35, 37]. Believing that they would be viewed as inferior, incapable, and unreliable, participants seemed to prefer job security over financial compensation and growth opportunities. Moloney et al. [38] refers to this as “going the extra mile”, an identity management technique commonly observed in working women with disabilities to combat stigmatic devaluation threats and prevent their “professional death”. As SLE mainly affects women, it should be a cause for concern that they are already faced with double binds in the workplace, where women carry higher expectations and punishments, and lower rewards and tolerance [32, 39]. Previous research has shown that women with SLE experience higher rates of WD than men [3, 11]. In fact, Moloney et al. [38] describes this double jeopardy to be a double bind through the compounding impacts of contradictory gender expectations and disability-related discrimination. A study by Kaptein et al. [32] found that women with arthritis disability report a greater unmet need for workplace accommodations despite reporting more activity limitations than men. The consequences of the COVID-19 pandemic (e.g. mental stress, vaccination, social distancing, public health guidelines), had also changed participants perspective on work engagement with many foregoing the benefits of in-person work for increased job control, self-preservation of health status and prevention of illness from the virus with remote work. They impact of the workplace environment and social support were even more so emphasized in the pandemic as they managed with the difficulties of their disease. Importantly, these challenges are modifiable and further education, awareness, and workplace accommodation availability is important to improve working conditions for those with SLE [40].

]To address this gender expectations, stigmatic devaluation, and gender-related discrimination, researchers should acknowledge the crossroads of the negative and positive impacts of WD and the disabling situations [34, 35]. Williams-Whitt et al. [42] challenge medical researchers to designing interventions that incorporate the principles and approaches of positive psychology and address disability by embracing it as a normal part of aging and the human condition. Focusing on the negative impacts allows the depiction of people with disabilities as burdens to employers and society to continue to propagate. However, a positive approach to WD would identify opportunities and capabilities that can be enhanced by reflecting on experiences with disability, rather than solely focusing on individual’s limitations, pain, and bodies [35, 41]. Studies of rheumatic diseases have shown that individuals with SLE prefer sedentary work and accommodations, which is consistent with the findings of this study [11]. Patient interviews were toward the end of the COVID-19 pandemic, and this could have impacted our findings on WD factors and accommodation use. Therefore, providing those with SLE improved job control, decision latitude/autonomy, sedentary accommodations, and reduced mental or physical demands may improve work outcomes and reduce WD.

SLE carries the potential for significant neuropsychological and psychological impact during work participation [42]. Neurocognitive impairments and discoid lupus have been associated with WD, with patients experiencing greater anxiety, worse mental health, and impaired visual memory, processing speed, and attention relative to non-disabled participants [12, 22]. Such impairments have greater prevalence in patients without WD than with WD, implying an existing cognitive burden associated with their work [44]. These patients also have higher levels of abnormal illness-related behaviours and helplessness, and lower levels of social support, self-efficacy, and self-reported physical and mental function [3, 11]. The uncertainty of SLE symptom onset and worsening can greatly affect an individual’s willingness and ability to engage with daily activities and social functioning leading to greater psychosocial stress [20]. Depression, anxiety, and stress contribute to greater WD, with more symptoms increasing the likelihood of experiencing WD [18, 23]. However, despite its high prevalence and impact, depression is infrequently identified and managed in rheumatology settings [44]. Further research is required to understand the longitudinal relationship between illness-driven activity limitations, accommodation use, and psychosocial factors to develop work interventions to support gainful employment in individuals with SLE and other rheumatic diseases.

Emerging research demonstrates that some multi-component interventions focusing on the prevention of WD may be more effective than the provision of work accommodations or job switching [3, 41, 45]. WD prevention interventions may be accomplished through multiple components including identification and modifications of work design, workplace or equipment, work environment, work conditions, colleague social support, and direct case management support with the worker and supervisor may be helpful for this population [35]. In addition, workplace resources that focus on accessibility of external rehabilitation supports for IADL management and supports outside of the workplace, including navigation through health service, worker’s compensation, and disability management systems may also be helpful [46]. For example, Keysor et al. [43] performed a randomized trial a WD prevention intervention for persons with musculoskeletal and rheumatic conditions. This intervention was implemented by rehabilitation workers (e.g. physiotherapists and occupational therapists) focused on identification of work barriers and development of subsequent supports through action planning, written employment supports, and telephone follows ups. They found no effect on work limitation but did observe reduced work loss. Therefore, WD prevention programs should consider multi-component interventions to improve their efficacy.

### Study limitations

Several limitations should be acknowledged in our study. The categorization of job activities into manual, sedentary, and mixed categories, while practical for the scope of this study, represents an oversimplification of the diverse nature of occupations. Doing so may not capture the nuanced demands of specific job roles and could limit the generalizability of our findings. Lack of information on socioeconomic status further restricts our understanding of the relationship between economic factors and WD in individuals with SLE. Participant experiences may lack generalizability to countries with different employment protections or social programs as well as geographic locations with higher representations of ethnic and multicultural persons with SLE. The absence of a healthy control group in this study is also a limitation, as it restricts the ability to directly compare the experiences of individuals with SLE to those without the condition. This study and the semi-structured interview questions were guided by WD prevention framework. The WD prevention framework does not posit that aspects of temporality influence WD. Consequently, it is possible that aspects of temporality such as the stage of life where SLE is experienced and the course of the disease itself, may not have evolved in the interviews. Future researcher may consider using other models such as the Disability Creation Process model in addition to the WD prevention framework to fully understand the experience of WD in individuals with SLE. Future studies may also benefit from the utilization of standardized instruments to procure objective measures of WD and accommodations, aiming for a more granular classification of job types and their respective impacts, and incorporating measures of socioeconomic status to provide a comprehensive analysis.

## Conclusion

With fatigue, physical pain, mental health challenges, and cognitive impairments being reported as the main aspects of WD in SLE patients, WD prevention will necessitate the recognition of the complexity, multidimensionality, and temporal aspects of SLE and its relationship to work. This study underscores the necessity of a collaborative, multidisciplinary intervention that extends beyond medical approaches to effectively mitigate influential psychosocial and workplace factors. A goal-oriented preventative framework to reduce WD in SLE patients would require enhancing work flexibility, refining regulations for accessing accommodations, and improving public education to diminish the prevailing stigma surrounding SLE. By embracing a holistic strategy, the ability to proactively address the unique challenges faced by SLE patients in the workplace and foster an environment conducive to their sustained employment and well-being can be enabled.

## Electronic supplementary material

Below is the link to the electronic supplementary material.

Supplementary Material 1

## Data Availability

The datasets used and/or analyzed during the current study are available from the corresponding author on reasonable request.

## Appendix A

***Interview Guide Protocol and Questions***

*Before the Interview with the Participant*

The session begins by making the participant comfortable through information conversation, including confirmation of their name and brief work history. Confidentiality issues are discussed, including informing participants that only the research team will know what the participant discusses, and that the discussion content will be combined with other focus group data for analysis. The interviewer will request permission to record the interview and explain the importance of recording for data collection accuracy. Participants will also be invited to provide their contact information if they wish to receive a one-page summary of the study findings upon completion of the project.

***Introduction to interview***

Returning to work with lupus can be challenging. To understand this process more fully, I am interested in listening to your experiences as a worker. There are no right or wrong answers to the questions. The questions relate to lived-experiences, views, thoughts, and feelings about going back to work.

1. What it is like to work (currently or in the past) with lupus?

Follow-up question: How has living with lupus impacted your physical and mental health? Please describe these impacts in whatever way is comfortable for you.

2. Tell me about your preferred area of work.

Follow-up question: Describe a typical workday for you?

3. Can you tell me about leaving your former job?

Follow-up question: What was your decision to leave work like?

4. What are some of your reasons for going back to work?
5. Describe the influences encouraging you to return to work.
6. Describe the challenges standing in your way of returning to work.
7. How is your quality of work-life?
  - If you could change something to improve your quality of work-life, what would it be?
8. Is there anything else we have not discussed that you would like to add?

**Thank you for your participation.**

## Abbreviations

SLE: Systemic Lupus Erythematosus
WD: Work Disability
PDQ-21: Perceived Deficits Questionnaire
WDP: Work Disability Prevention
NSAIDs: Nonsteroidal anti-inflammatory drugs
